# Annual Baseline King-Devick Oculomotor Function Testing Is Needed Due to Scores Varying by Age

**DOI:** 10.3390/sports9120166

**Published:** 2021-12-13

**Authors:** Dearbhla Gallagher, Doug King, Patria Hume, Trevor Clark, Alan Pearce, Conor Gissane

**Affiliations:** 1 Human Performance Exercise & Wellbeing Centre, Bucks New University, Bucks, Buckinghamshire HP11 2JZ, UK; Dearbhla.Gallagher@bucks.ac.uk; 2 Sports Performance Research Institute New Zealand (SPRINZ), Faculty of Health and Environmental Science, Auckland University of Technology, Auckland 1142, New Zealand; phume@aut.ac.nz; 3 School of Science and Technology, University of New England, Armidale, NSW 2351, Australia; 4 Wolfson Research Institute for Health and Wellbeing, Department of Sport and Exercise Sciences, Durham University, Durham DH1 3LE, UK; 5 National Institute of Stroke and Applied Neuroscience (NISAN), Faculty of Health and Environmental Science, Auckland University of Technology, Auckland 1142, New Zealand; 6 International College of Management, Sydney, NSW 2095, Australia; tclark@icms.edu.au; 7 College of Science, Health and Engineering, La Trobe University, Melbourne, VIC 3086, Australia; Alan.Pearce@latrobe.edu.au; 8 School of Sport Health and Applied Science, St. Mary’s University, Twickenham, London TW1 4SX, UK; conor.gissane@btinternet.com

**Keywords:** oculomotor function, internal consistency, test–retest reliability, individualised, baseline testing

## Abstract

Objective: To document baseline King-Devick (K-D) oculomotor function scores for male and female participants aged between 4 and 20 years old. Methods: Utilising a cross section of schools, rugby clubs and gymnastic clubs, 1936 participants (1300 male, 636 female) completed the spiral-bound K-D test for the identification of disturbed oculomotor function. Results: This study identified that overall, the baseline scores of the K-D test became faster by 1.4 (0.3 to 4.5) s per year, when compared with the previous age group in the same number of reading card groups. When comparing normative values of the original K-D validation study with the same age groups of the current cohort, participants aged 6 to 11 years recorded a faster baseline time (range 3.5 to 8.6 s), while those in the 12 to 14 years. age group recorded slower baseline times (range −3.9 to −7.9 s). Discussion: In general, there were age group differences, but not sex differences, for K-D test times in the current cohort. Analysis of single card times, across all age groups, showed changes likely due to improved reading time. Conclusion: The results support the need for individualised annual pre-injury baseline testing of the K-D test.

## 1. Introduction

In recent years, awareness of concussion has grown [1] with increasing attention being paid to professional and elite sport, where athletes are closely monitored and medical support teams are well established. Given the health risks associated with concussion [2], challenges arise for the clinicians, coaches, and parents who are responsible for those playing amateur and junior-level sport. This is magnified by the ambiguity in the presentation and pathophysiology of concussion in an immature brain when compared to a mature brain [3]. Available resources can vary in terms of the medical provision, associated costs, and time availability for the testing and assessment of anyone with a concussion. Concussion awareness and education campaigns have emerged, aiming to enlighten those involved in sport to recognise and remove a player with suspected concussion from play. The next step in the identification and assessment of concussion is to provide coaches, parents, and clinicians, with easily available tools to identify concussion whilst assisting in the guidance of recovery from the effects of a concussion and enabling a return to participation in the athletes’ chosen activities. 

The Standardized Concussion Assessment Tool version 5 (SCAT5) and ChildSCAT5 are freely downloadable tools for concussion evaluation [4]. The SCAT5 and ChildSCAT5 comprise a series of assessment tools for concussion, including a sideline assessment, symptom evaluation (Post-Concussion Symptom Score), cognitive tests (orientation, memory, balance, and coordination), and a balance examination [5]. These assessment tools are reviewed on a four-yearly cycle through the Concussion in Sport Conference (CISC). Such assessments are also endorsed by major sports organizations such as the International Olympic Committee, World Rugby, and Fédération Internationale de Football Association (FIFA). The most recent rendition is the 5th version published after the 2016 CISC [4,5,6]. Although the SCAT5 and ChildSCAT5 present the most recent concussion assessment tool, there are several limitations associated with their use. The time it takes to complete the SCAT5, and the need for medical personnel means this is more of a training room or clinical assessment tool rather than a sideline assessment tool [7]. In addition, there is a notable absence of the assessment of oculomotor capacity in both the SCAT5 and ChildSCAT5, yet research indicates that there is a need for an oculomotor screening examination [8,9] in order to provide objective physiological evidence that a concussion has occurred [10]. 

Concussive, and sub-concussive, impacts to some areas of the brain can cause neurological changes that may go undetected on tests of both cognitive and locomotor function [11]. Disturbed oculomotor function is a common symptom in many concussions [9,10]; therefore, evaluating this aspect provides another valuable parameter in the assessment of concussion [9]. Vitally, oculomotor function affects over half of the brain’s pathways [12] that can deteriorate following a concussive injury [13]. The King-Devick (K-D) test is an oculomotor tool that can be utilized in the assessment of concussion through the identification of disturbed oculomotor function. The K-D has previously been reported to both be a reliable [14,15] and valid [12,16] concussion assessment tool. To evaluate oculomotor integrity and function, those with a suspected concussion read a series of numbers as fast as possible from three test cards with increasing difficulty depending upon their age. The K-D test has several benefits including cost effectiveness, brevity, ease of use and the ability to be utilized by non-medical professionals [17]. These factors are particularly valuable in non-elite sport where medical care is often limited or non-existent. The K-D compliments the SCAT3 and has been proposed to improve the sensitivity of concussion detection by up to 100% when utilized with components of the SCAT3 [5,6,7].

Changes that occur because of a concussive injury are best assessed by the detection of deviations from annual pre-injury baseline values, thus affording the concussion test increased objective ability [4]. Subjective information, which is frequently relied upon in the assessment of concussion, is often poorly informed and at any time may be unreliable [18,19]. In contrast, the K-D is based on timed objective performance of oculomotor speed and precision [12]. Symptom reporting and generalised guidelines are currently utilised for return to play [20]. However, the K-D can be utilised immediately in injury assessment [12,21] and during both recovery and return to play [16,21]. To assist with this, it is recommended that annual pre-injury baselines be conducted for all participants to enable a direct comparison between the post-injury and the pre-injury baseline. In the absence of baseline scores, it has been reported that any change in performance on the initial post-injury K-D score may either be reflective of the participants usual performance, or be attributed to a concussion having occurred [22]. To assist with clinicians’ assessment of concussion with participants with no recorded baselines, some studies have reported K-D normative data [22,23]. This is despite the recommendation of annual pre-injury baseline assessments being conducted, as comparison of K-D score results should be a direct comparison with the individual’s baseline only. Although numerous studies have reported on the K-D in concussion assessment, no study to date has reported on the differences between age groups on the scores of the K-D to identify the need for annual baseline assessments. Therefore, the purpose of this study was to document the baseline scores of male and female participants from 4 to 20 years old to enable the identification of differences in K-D oculomotor function score due to age group and sex.

## 2. Methods

### 2.1. Participants

Using a cross section of schools (athletic and non-athletic students), rugby clubs and gymnastic clubs based in the United Kingdom, 1936 participants (aged 4 to 20 years; 636 females and 1300 males) completed this study. Those who were recovering from a concussive injury were excluded from participating in the study. Data collectors were qualified and/or student athletic trainers, sports rehabilitators and/or support staff trained in the use of K-D. Data collection was always completed in the presence of the lead researcher. No other exclusions were made for participation and the primary language used was English. Parent/guardian consent was gained with participant and parent information sheets and exclusion forms provided. The exclusions identified were: (1) in cases of a visual impairment where participants would normally have a corrective prescription for glasses or contacts, but did not have access to it at the time of testing; (2) a suspected concussive incident in the previous three months that had not been medically examined; (3) where a participant had not returned to sport following a concussion; and (4) where the test could not be completed in English or where English was not the first language of a participant. All procedures were approved by the ethics committee (SMEC 2014-5 037) of the lead authors’ institution. 

### 2.2. King-Devick Test

The King-Devick test is a saccadic test measuring the speed of rapid number naming [24]. Participants were asked to read the numbers on each card aloud from left to right as quickly as possible without making any mistakes. The time taken for each card was recorded as was the number of reading errors made and this was combined to provide a summary score for the entire test (the K-D test score). The entire test took less than two minutes to administer per participant. The K-D test has been reported to have an inter-class correlation for test–retest reliability of 0.96 [17] and 0.97 [15]. The spiral-bound flip card K-D test (V2.0.0) was utilised for the study. 

### 2.3. Data Collection Procedures

The K-D test involves getting participants to read aloud a series of random single-digit numbers from left to right. The K-D test included one practice (demonstration) card and three test cards on a spiral-bound moisture-proof 6 × 8-inch physical test (www.kingdevicktest.com, 7 December 2021). As per standardised instructions of the test, participants aged 5–7 years completed the first card only; those aged 8–9 years completed cards 1 and 2; and those aged 10 years and above completed all three cards. Participants aged 4 were also included in the study to enable identification of the differences with the 5 years-old participants.

### 2.4. Procedures

Testing took place in the given practice area for each sport, training room and class group. Tests could only be completed if the child had not taken part in any athletic activity in the previous 30 min, to avoid any potential confounding effects from physical exercise or fatigue [25]. Testing was completed by trained personnel. Each participant was given standardised instructions documented on the demonstration page of the K-D test. The tester provided an example of how to complete the test using the demonstration card. Using a digital stopwatch, testing began when the participant began reading the first number on each test page and finished on calling the last number of the test card. The total time for the test was recorded including any errors made that were not self-corrected. Each test paged time was recorded and those reading two or more test cards had their times summed. Two trials of each test card were completed, with the fastest score completed without any reading errors taken as the participant’s baseline. 

### 2.5. Statistical Analyses

**Figure 1:**
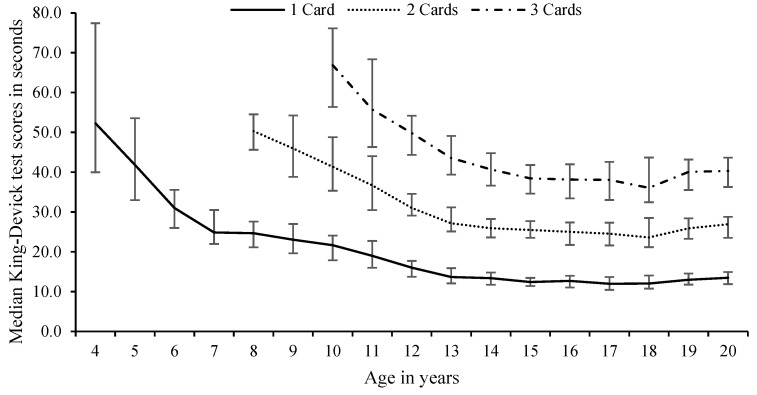
Differences in median scores with inter-quartile ranges of all the King-Devick test cards by age of participants and the number of number of cards read for combined participants aged 4 to 20 years. Participants aged 4–7 years read 1 card, 8–9 years read two cards, and 10+ years read three cards. The lower solid line is the time for 1 card only, as the actual card time for 4–7 years, the fastest single card time of the 2 cards read for the 8 to 9 years, and the fastest single card time of the 3 cards read for the 10 to 20 years.

**Figure 2:**
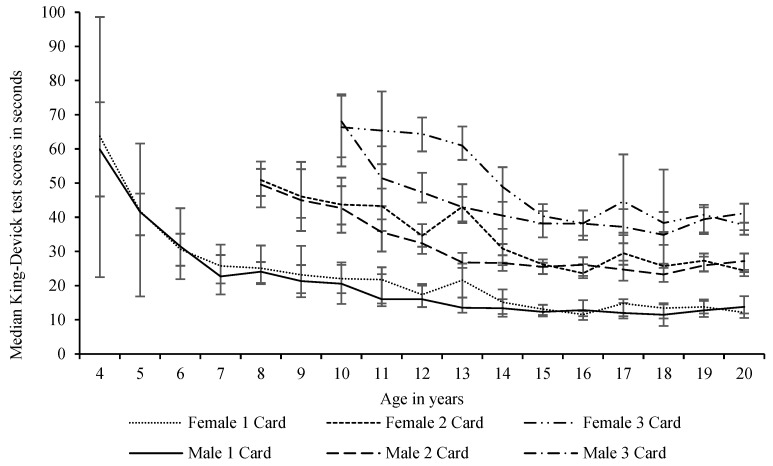
Differences in median scores with inter-quartile ranges of all the King-Devick test cards by age and gender of participants and the number of cards read for combined participants aged 4–20 years Participants aged 4–7 years read 1 card, 8–9 years read two cards, and 10+ years read three cards.

Participants aged 4–7 years read 1 card, 8–9 years read two cards, and 10+ years read three cards. To compare test card scores between the ages, the 1-card time for 4–7 years olds, the fastest single-card time of the 2 cards read for the 8 to 9 years olds, and the fastest single-card time of the 3 cards read for the 10 to 20 years olds were recorded as the 1-card time for combined genders (see Figure 1) and for each gender across the ages reported (see Figure 2). 

All K-D test scores were entered into a Microsoft Excel spreadsheet and analysed with SPSS (V25.0 Armonk, NY, USA: IBM Corp). Data were checked for normality and homogeneity of variance using Shapiro–Wilk’s test (*W*_(1649)_ = 0.78; *p* < 0.0001) and a one-sample *t*-test (*t*_(1648)_ = 89.0; *p* < 0.0001). The testing scores were evaluated, and the baseline was identified for males, females and sexes combined. The resulting scores were analysed with a Friedman repeated-measures ANOVA on ranks. If any notable differences were observed, a Wilcoxon signed-rank post hoc test was conducted with a Bonferroni correction applied. The differences between the established baselines were identified and a one-sample *t*-test was utilised to analyse this across the age groups by female, males and sexes combined for 1 card, 2 card, 3 card and all cards. Cohen’s [26] effect size (*d*) was utilised to calculate practically meaningful differences between the different age groups, males and females and the different trials. Effect sizes of <0.19, 0.20–0.60, 0.61–1.20 and >1.20 were considered trivial, small, moderate, and large, respectively [27]. Internal consistency reliability for the test cards vs. total times scores were measured using Cronbach’s alpha (α). Test–retest reliability was estimated utilising the intra-class correlation coefficient (ICC), with 95% CI, to examine agreement between first and second baseline test scores. The level of significance was set at *p* ≤ 0.05, and all data were expressed as medians with interquartile (25th–75th) range, except where stated.

## 3. Results

**Figure 3:**
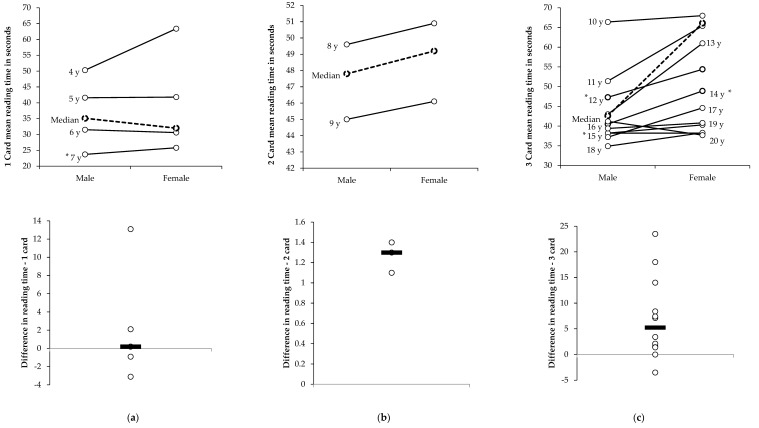
Median reading times and differences in times for the King-Devick test baseline scores for male and female participants aged 4 to 20 years by number of test cards. Bold broken line is median time for number of cards. Thick line is mean difference between male and female. * = Significant difference (*p* < 0.05) between male and female times. (**a**) 1 card age 4–7 years; (**b**) 2 cards age 8–9 years; (**c**) 3 cards age 10–20 years.

The mean and standard deviation for age of the cohort was 10.7 ± 4.6 years (males: 11.7 ± 4.7 years; females: 8.4 ± 3.5 years) and ranged from 4 to 20 years old. A list of the median and interquartile ranges of the participants by age are shown in Figure 3. For the one-card category, the median K-D test times of participants in the 6 years-old group (31.0 (26.0 to 35.6)) s) were faster when compared with the 5 years-old group (41.8 (33.0 to 53.6) s; *χ*^2^_(1)_ = 36.5; *p* < 0.0001; z = −6.6; *p* < 0.0001; *d* = 0.88). For the two-card category, the median K-D test times in the 8 years-old group were longer (50.5 ((45.5 to 54.7)) s) when compared with the 7 years-old group (24.9 (22.0 to 30.5) s; *χ*^2^_(1)_ = 13.66; *p* < 0.0001; z = −10.1; *p* < 0.0001; *d* = 1.64) as the number of cards increased from one card to two cards. The increase in cards read time difference from two in the 9 years group to three in the 10 years group lead to similar longer times for the 10 years-old group (70.9 (60.0 to 81.3) s) when compared with the 9 years-old group (45.5 (38.8 to 54.1) s; *χ*^2^_(1)_ = 97.2; *p* < 0.0001; z = −9.0; *p* < 0.0001; *d* = 1.56). The median K-D test times for the males (47.2 (40.2 to 51.0) s) were faster than the females (79.3 (75.5 to 83.7) s; *χ*^2^_(1)_ = 8.9; *p* = 0.0039; z = −3.0; *p* = 0.0029; *d* = 4.39) in the 12 years-old age group. There were no differences between the sexes for any other age groups.

When grouped together with the number of cards read, the males recorded a slower median K-D test time for test 1, (36.0 (27.4 to 51.3) vs. 33.1 (27.0 to 43.2) s; *χ*^2^_(1)_ = 6.9; *p* = 0.0088; z = −3.8; *p* = 0.0002; *d* = 0.16), test 2 (38.6 (30.5 to 56.8) vs. 35.4 (29.1 to 50.0) s; *χ*^2^_(1)_ = 5.8; *p* = 0.0162; z = −3.7; *p* = 0.0002; *d* = 0.18), and for the baseline K-D test time (35.1 (26.0 to 48.6) vs. 32.0 ((26.1 to 42.0) s; *χ*^2^_(1)_ = 5.5; *p* = 0.0188; z = −3.7; *p* = 0.0002; *d* = 0.16) when compared with females using one card (ages 4 to 7 years old) (see Table 1). There was a slower median time recorded for the second trial for the one-card reading group for males (38.6 (30.5 to 56.8) vs. 36.0 (27.4 to 51.3) s; *χ*^2^_(1)_ = 31.2; *p* < 0.0001; z = −3.7; *p* = 0.0002; *d* = 0.14), females (35.4 (29.1 to 50.0) vs. 33.1 (27.0 to 43.2); *χ*^2^_(1)_ = 32.6; *p* < 0.0001; z = −3.8; *p* = 0.0002; *d* = 0.13) and combined (37.1 (29.7 to 52.5) vs. 34.0 (27.0 to 47.1) s; *χ*^2^_(1)_ = 63.1; *p* < 0.0001; z = −9.5; *p* < 0.0001; *d* = 0.13) when compared with the first trial. This was similar for the two-card reading group (age 8 and 9 years old) for males (54.3 (46.7 to 67.1) s vs. 48.6 (41.2 to 56.3) s; *χ*^2^_(1)_ = 90.1; *p* < 0.0001; z = −6.7; *p* < 0.0001; *d* = 0.63), females (53.6 (48.4 to 58.6) vs. 49.8 (43.9 to 54.8) s; *χ*^2^_(1)_ = 53.0; *p* < 0.0001; z = −6.9; *p* < 0.0001; *d* = 0.47) and combined (54.1 (48.0 to 62.2) vs. 49.4 (42.6 to 55.3) s; *χ*^2^_(1)_ = 138.6; *p* < 0.0001; z = −12.2; *p* < 0.0001; *d* = 0.55). 

In the three-card reading group there was a slower median time for the second (70.0 (65.5 to 74.2) s) when compared with the first (66.4 (51.2 to 79.2) s; *χ*^2^_(1)_ = 44.0; *p* < 0.0001; z = −5.8; *p* < 0.0001; *d* = 0.47) trial time for female participants (see Table 1). Males in the three-card reading group recorded a faster median time in the second (40.6 (35.3 to 45.9) s) when compared with the first trial (44.4 (38.8 to 53.3) s; *χ*^2^_(1)_ = 78.0; *p* < 0.0001; z = −10.1; *p* < 0.0001; *d* = 0.58). When comparing the fastest and slowest K-D test time for participants reading the three cards (age 10+ years) there were differences observed for males (−2.1 (−3.7 to −0.9) s; *χ*^2^_(1)_ = 78.0; *p* < 0.0001; z = −10.1; *p* < 0.0001; *d* = 0.21), females (−2.6 (−4.6 to −1.3); *χ*^2^_(1)_ = 44.0; *p* < 0.0001; z = −5.8; *p* < 0.0001; *d* = 1.12), and for the combined scores (−2.1 (−3.8 to −0.9); *χ*^2^_(1)_ = 95.4; *p* < 0.0001; z = −10.9; *p* < 0.0001; *d* = 0.34). The Cronbach’s α varied between 0.864 (two cards, combined sexes) and 0.996 (three cards, male) for the internal consistency of the tests completed. The ICC varied between 0.782 (2-card male) and 0.996 (three cards, female) for the test–retest reliability of the tests completed. 

As can be seen by Table 2, on average, females recorded a faster change in the median baseline K-D test when compared with the previous age group for one-card (−1.6 s vs. −0.8 s; *p* = 0.7036; *d* = 0.14), two-card (−2.8 vs. −1.4 s; *p* = 0.8660; *d* = 0.32), and three-card (−2.6 vs. −2.3 s; *p* = 0.9407; *d* = 0.55) testing when compared with males. When all the test cards were combined, females recorded a faster change in the median baseline K-D test when compared with the previous age group than males recorded (−2.0 vs. −1.4 s; *p* = 0.8516; *d* = 0.04).

## 4. Discussion

The K-D oculomotor function test can assist in the rapid identification of concussion. The use of baseline data specific for an individual on the sideline can enhance the ability to detect any changes that occur from a concussive injury. This study undertook documenting the baseline scores of participants ranging in age from 4 to 20 years to enable the identification of differences due to age group and sex. This is one of the largest studies on the topic to date, with 1936 participants over a 16 years age span. Although previous studies reporting on the K-D test have included pre- and post-injury scores [12], and some studies have endeavoured to report on normative data for specific playing populations [22,23], no study has reported K-D scores for differences in ages over a large cohort. 

This study identified that overall, the baseline scores of the K-D test became faster by a median of 1.4 (0.3 to 4.5) s per year, when compared with the previous age group in the same number of reading card groups. This is similar to other studies reporting 0.3 s to 2.9 s improvement in time with every 1 years increase in age. This increment highlights the finding that baseline tests for the K-D test should be undertaken annually.

Although the use of normative data for K-D tests has been previously reported for male ice hockey [23] and high school American football players [22], it has also been reported [28] that to use the K-D test without a baseline to compare results, there appears to be a reported weak sensitivity and specificity when assessing for a concussive injury. The original validation [29] of the K-D test was for reading eye movement disorders and, when compared to a similar age group (age 6 to 14 years) study [30] in another country, it was identified that there were differences in the normative ranges of these same groups. This may be the same when utilising normative data from different sporting environments and countries. In comparing the normative data, it was identified that academic influences in visual development may have contributed to the results [30]. In the original study, participants commenced formal education as young as 4 years old, whereas in the comparison study [30] this occurred at age 6 years It has also been reported that there is a negative correlation (*r* = −0.194; *p* = 0.002) between K-D time and education level, where the higher the education level, the faster the K-D time was recorded [31]. As the normative data that have been previously reported [22,23] do not report differences in education level, and not all countries have the same academic influences, then the utilisation of normative data has its limitations. This highlights the problem with utilising normative data for comparisons in the assessment of a concussive injury. Although all the participants in the current study inclusive to the age of 17 years were in formal education, there were no separate analysis conducted for specifics such as academic achievement and level of education. As such, the K-D times reported in this study should not be utilised as the validation of normal ranges of scores for the K-D test for participants aged 4 years to 20 years

Normative data have been utilised extensively in other fields where baseline and pre-injury testing are not feasible [32]. This may be due to limited resources available, the facilities utilised and time factors imposed for the assessment to be conducted [33]. It is possible that, when comparing post-concussion scores to normative values derived from a different sample, the values utilised could influence impairment identification resulting in returning the athlete to participation prematurely [33]. Due to the intricacy of concussion assessment, the use of normative data may be inaccurate as the values derived have been reported to be affected by education level [34], history of attention-deficit/hyperactivity disorder, low/high intelligence, learning disabilities [35,36,37], socioeconomic status [38], race [39], history of concussion [33,40,41] and the sport played [34,42]. It has been reported [43] that factors such age, sex, and history of concussion may be risk factors for influencing the results of the K-D test but there is a paucity of studies reporting on the other aspects identified that could also influence the results of the K-D test. As a concussed athlete’s post-injury performance would be below the normative values, any retesting results will likely see practice effects on subsequent retesting, necessitating retest normative data to be established [32]. Therefore, although the use of normative data for post-injury evaluation is attractive as it less time and resource intensive, in terms of concussion it does not have the research base established for its use in the field of concussion assessment and management [32]. 

The findings reported here were obtained on the spiral-bound book version of the K-D test. Although this testing platform is identical to the original study [29], this version is no longer available. The current K-D test is available for use on a tablet or iPad-based platform and consists of three versions of the original K-D test. When comparing the differences in the times recorded on the spiral-bound with the computerised versions of the K-D test, there were statistically significant (*p* < 0.0001) differences recorded [44]. If the baseline K-D test was completed on the iPad-based platform, and the spiral-bound platform was utilised for concussion, a concussive injury may be missed due to the spiral-bound version being faster [44]. On average, there was a 3.7 s difference between these two testing platforms and this may have clinical implications [44]. In a recent meta-analysis [12], it was reported that there was a mean worsening of K-D time of 4.8 s (range 3.7 to 5.8 s) from baseline in concussed participants. The differences reported between the different platforms may result in a concussed player being inadvertently returned to activity. 

There is an increasing body of literature [12] reporting the use of the K-D test in the sporting environment for the identification of concussion. As concussion is a particularly difficult injury to identify and manage, the use of a tool such as the K-D test as part of a continuum of assessment tools for concussion in the initial sideline assessment for a concussion can assist in the rapid identification and removal of an athlete. As it is not possible to readily identify a concussion, utilising an assessment tool without any baseline data for the identification of concussion is limited as any result obtained may be particular to the individual being assessed, or may in fact indicate a decline from their normal status [23]. The use of baseline data specific for that individual on the sideline would enhance the ability to detect any changes that occur from a concussive injury. This would aid in the removal of the participant from further participation and the subsequent risk of a potentially more catastrophic injury [2]. 

There is a learning effect with the repeated use of the K-D test [12] especially within shortened testing timeframes [45]. This may have been more apparent in the resultant scores by age groups if the participants in this study were tested repeatedly over subsequent years, but this was not the case. The participants here were not longitudinally followed, so the learning effect did not have any influence on the scores of subsequent age years for the K-D test. As a result, the differences between the subsequent years of age for the K-D test scores show a faster time recorded with a small effect size (*d* = 0.55). The changes observed in reading time by age group further support the need for annual baseline pre-injury testing of the K-D test and not to utilise normative-based data for the evaluation of post-injury K-D scores in a suspected concussive injury.

As can be seen in Figure 1, the K-D test scores decreased with age as the participants got older within the relevant number of card age groups. As the number of cards increased (i.e., age 7 to 8 years old: one to two cards and age 9 to 10 years old: two to three cards), initially the times became slower but with increasing age began to get faster. The changes observed with the increases in age, and improved K-D times, have been suggested to occur as a result of the developmental changes happening in saccadic eye movements and cognition [8,30]. It has been previously suggested that the improvement in these times was due to the development of white and grey matter areas of the frontal lobes and the time improvement towards this stabilising effect is largely due to the shortening of saccade reaction times, or latency [12]. Interestingly, some age groups recorded a slower median K-D test time than the previous age group, i.e., female 12 vs. 13 years old age group (66.1 s vs. 79.3 s), despite using the same number of testing cards. The reason for the slowing of reading times by the female group may be related to differences in academic influences, developmental saccadic eye movements and cognition [8] and highlights the non-use of normative profiles for the use of assessment with the King-Devick. Further research is warranted to explore if this finding occurs on other cohorts of female participants of different ages.

In a recent study [46], participants under the age of 13 years were only tested using the two-card assessment. By adjusting the results of the current study to report the median scores to only two cards at age 10 to 12 years (see Figure 1), there were notable improvements by each age group. In addition, when the participants commenced the three-card assessment K-D test, the median time difference was not so pronounced. The study [46] did not report any concussive injuries in the 9 years to 11 years age group but did report that concussive injuries were identified in the 12 years age group using the two-card K-D test assessment. As there were no other studies reporting on the use of two cards for the 10 years to 12 years age group, further research is warranted to identify the differences in sensitivity and specificity and in the assessment of concussion by changes in each test card.

Limitations to this study were that not every age group had the same number of participants and an equal distribution of males and females. This may have resulted in variations in the median scores reported. Although participants were asked about any known learning or visual difficulties prior to taking part, there were no formal investigation undertaken to verify the responses of the participants, or their caregivers who consented to participate in the research. For those that did report any known learning or visual difficulties, no further analysis was conducted. There was no verification of education level, history of attention-deficit/hyperactivity disorder, high/low intelligence, learning disabilities, socioeconomic status, ethnicity, sports participation, or history of concussion. Further research should consider utilising the same number of males and females and, if comparing different age groups, the same number of participants. Further research should also be considered for differences in baselines between participants with and without a history of concussion. 

## 5. Conclusions

This study identified that overall, the baseline scores of the K-D oculomotor function test became faster (improved) by 1.4 (0.3 to 4.5) s per age group. Therefore, the use of annual pre-injury baselines is recommended. In general, there were no sex differences by age group for K-D test times in the current cohort.

## Figures and Tables

**Table 1:** King-Devick test scores for male and female participants aged 4 to 20 years, and for sexes combined, for the number of cards for the age group category, shown by median with interquartile ranges for trials 1 and 2, differences between trials 1 and 2, baseline/fastest versus slowest test score results, Cronbach’s alpha, and interclass correlation.

			Trial 1 (s)	Trial 2 (s)	Difference Trial 1 vs. Trial 2 (s)	Baseline/Fastest Times (s)	Slowest Times (s)	Difference Fastest vs. Slowest (s)	Test–retest and Cronbach’s α between Trial 1 and Trial 2	
		*n*=	Median (IQR)	Median (IQR)	Median (IQR)	Median (IQR)	Median (IQR)	Median (IQR)	α=	ICC (95% CI)
1 Card (4–7 years)	Male	336	36.0 (27.4–51.3) ^bd^	38.6 (30.5–56.8) ^ad^	2.2 (−1.5–10.0)	35.1 (26.0–48.6) ^df^	41.2 (31.4–61.2) ^de^	−5.0 (−11.0–−2.0)	0.946	0.940 (0.911–0.957)
	Female	290	33.1 (27.0–43.2) ^bc^	35.4 (29.1–50.0) ^ac^	2.8 (−1.4–7.0)	32.0 (26.1–42.0) ^cf^	36.7 (30.3–50.8) ^ce^	−4.4 (−8.6–−1.9)	0.961	0.957 (0.938–0.969)
	Combined sexes	626	34.0 (27.0–47.1) ^b^	37.1 (29.7–52.5) ^a^	2.4 (−1.4–8.4)	33.1 (26.1–45.1) ^f^	38.6 (31.0–54.7) ^e^	−4.5 (−10.0–−2.0)	0.955	0.950 (0.929–0.963)
2 Cards (8 and 9 years)	Male	130	48.6 (41.2–56.3) ^b^	54.3 (46.7–67.1) ^a^	6.2 (3.2–15.4)	47.8 (41.2–56.0) ^f^	57.0 (47.2–67.1) ^e^	−6.2 (−15.4–3.9)	0.871	0.782 (0.218–0.908)
	Female	146	49.8 (43.9–54.8) ^b^	53.6 (48.4–58.6) ^a^	4.7 (1.0–8.4)	49.2 (43.5–54.1) ^f^	54.5 (49.4–59.1) ^e^	−4.9 (−8.4–−2.1)	0.867	0.818 (0.549–0.907)
	Combined sexes	276	49.4 (42.6–55.3) ^b^	54.1 (48.0–62.2) ^a^	5.4 (1.9–11.0)	49.0 (42.1–54.4) ^f^	54.6 (48.4–62.3) ^e^	−5.5 (−11.2–−2.7)	0.864	0.796 (0.413–0.902)
3 Cards (10+ years)	Male	834	44.4 (38.8–53.3) ^b^	40.6 (35.3–45.9) ^a^	1.3 (−0.5–3.2)	42.9 (37.2–48.3) ^f^	42.7 (39.6–51.7) ^e^	−2.1 (−3.7–−0.9)	0.996	0.950 (0.921–0.966)
	Female	200	66.4 (51.2–79.2) ^b^	70.0 (65.5–74.2) ^a^	2.1 (0.2–4.1)	66.1 (51.2–79.2) ^f^	72.2 (69.1–76.4) ^e^	−2.6 (−4.6–−1.3)	0.957	0.996 (0.995–0.997)
	Combined sexes	1034	46.5 (39.7–58.1) ^b^	40.5 (36.0–45.7) ^a^	−1.4 (−3.4–0.4)	40.5 (35.6–45.7) ^f^	46.5 (39.7–58.1) ^e^	−2.1 (−3.8–−0.9)	0.955	0.948 (0.915–0.965)
Total Score	Male	1300	43.7 (36.3–53.3)	41.5 (34.6–50.5) ^d^	0.0 (−2.7–3.7)	42.0 (35.0–52.3) ^f^	43.8 (36.7–53.4) ^de^	−3.0 (−6.2–−1.3)	0.900	0.894 (0.881–0.907)
	Female	636	45.7 (33.6–62.0) ^b^	42.7 (33.0–54.7) ^ac^	2.6 (−1.4–7.0)	45.0 (32.4–60.0) ^f^	44.0 (34.0–55.9) ^ce^	−4.3 (−8.3–−2.0)	0.953	0.951 (0.942–0.959)
	Combined sexes	1966	44.0 (35.5–55.0) ^b^	41.9 (34.0–52.6) ^a^	0.6 (−2.2–5.3)	42.6 (34.3–54.0) ^f^	43.6 (35.6–53.9) ^e^	−3.3 (−7.0–−1.5)	0.917	0.913 (0.904–0.921)

Negative numbers indicate an increase (faster) change; IQR = interquartile (25th and 75th) range; s = seconds; α = Cronbach’s alpha; ICC = interclass correlation; CI = confidence interval; significant difference (*p* < 0.05) that (a) = Trial 1; (b) = Trial 2; (c) = male; (d) = female; (e) = fastest time; (f) = slowest times; baseline score is the fastest time completed without any reading errors of the two trials.

**Table 2:** Median and interquartile range summaries and the range of differences in the established baseline K-D test scores from previous ages by the number of cards read for females, males, and sexes combined for participants aged 4 years to 20 years.

	No of Cards	Differences from	Range of Differences (s)	Differences	
		Previous Age (s)		across Ages	
		Median (IQR)	Min–Max	*t=*	*p=*
Female	1 card	−1.6 (−4.8–−0.4)	−21.9–4.2	−2.1	0.0535
	2 card	−2.8 (−4.8–1.1)	−12.3–8.5	−1.3	0.2069
	3 card	−2.6 (−6.9–−0.1)	−12.1–6.4	−1.7	0.1208
	All cards	−2.0 (−4.8–−0.3)	−21.9–8.5	−3.0	0.0053
Male	1 card	−0.8 (−4.1–0.4)	−18.3–1.4	−2.2	0.0466
	2 card	−1.4 (−4.2–0.4)	−7.1–2.6	−2.3	0.0457
	3 card	−2.3 (−4.2–0.4)	−16.6–4.5	−1.5	0.1632
	All cards	−1.4 (−4.2–0.0)	−18.3–4.5	−3.4	0.0016
Sexes Combined	1 card	−1.2 (−2.9–−0.1)	−10.8–0.9	−2.7	0.0176
	2 card	−1.1 (−4.5–−0.4)	−5.7–2.3	−2.6	0.0237
	3 card	−2.1(−6.0–0.0)	−11.2–4.0	−2.0	0.0786
	All cards	−1.4 (−4.5–−0.3)	−11.2–4.0	−4.2	0.0001

IQR = interquartile (25th to 75th) range; negative numbers indicate an increase (faster) change.

## Data Availability

The data are not publicly available due to the manufacturer’s requirement for annual baseline assessments to be conducted and not normative ranges.

## References

[1] Khurana V.G., Kaye A.H. (2012). An overview of concussion in sport. J. Clin. Neurosci..

[2] King D., Brughelli M., Hume P., Gissane C. (2014). Assessment, Management and Knowledge of Sport-Related Concussion: Systematic Review. Sports Med..

[3] Davis G., Purcell L. (2013). The evaluation and management of acute concussion differs in young children. Br. J. Sports Med..

[4] McCrory P., Meeuwisse W., Dvořák J., Aubry M., Bailes J., Broglio S., Cantu R.C., Cassidy D., Echemendia R.J., Castellani R.J. (2017). Consensus statement on concussion in sport—The 5th international conference on concussion in sport held in Berlin, October 2016. Br. J. Sports Med..

[5] Echemendia R.J., Meeuwisse W., McCrory P., Davis G.A., Putukian M., Leddy J., Makdissi M., Sullivan S.J., Broglio S.P., Raftery M. (2017). The Sport Concussion Assessment Tool 5th Edition (SCAT5): Background and rationale. Br. J. Sports Med..

[6] Davis G.A., Purcell L., Schneider K.J., Yeates K.O., Gioia G.A., Anderson V., Ellenbogen R.G., Echemendia R.J., Makdissi M., Sills A. (2017). The Child Sport Concussion Assessment Tool 5th Edition (Child SCAT5): Background and rationale. Br. J. Sports Med..

[7] Eckner J., Kutcher J. (2010). Concussion symptoms scales and sideline assessment tools: A critical literature update. Curr. Sports Med. Rep..

[8] Galetta K., Morganroth J., Moehringer N., Mueller B., Hasanaj L., Webb N., Civitano C., Cardone D.A., Silverio A., Galetta S.L. (2015). Adding vision to concussion Testing: A prospective study of sideline testing in youth and collegiate athletes. J. Neuroophthalmol..

[9] Marinides Z., Galetta K.M., Andrews C.N., Wilson J.A., Herman D.C., Robinson C.D., Smith M.S., Bentley B.C., Galetta S.L., Balcer L.J. (2015). Vision testing is additive to the sideline assessment of sports-related concussion. Neurol. Clin. Pract..

[10] Echemendia R.J., Broglio S.P., Davis G.A., Guskiewicz K.M., Hayden K.A., Leddy J.J., Meehan W.P., Putukian M., Sullivan S.J., Schneider K. (2017). What tests and measures should be added to the SCAT3 and related tests to improve their reliability, sensitivity and/or specificity in sideline concussion diagnosis? A systematic review. Br. J. Sports Med..

[11] Talavage T.M., Nauman E.A., Breedlove E.L., Yoruk U., Dye A.E., Morigaki K.E., Feuer H., Leverenz L.J. (2014). Functionally-Detected Cognitive Impairment in High School Football Players without Clinically-Diagnosed Concussion. J. Neurotrauma.

[12] Galetta K.M., Liu M., Leong D.F., Ventura R.E., Galetta S.L., Balcer L.J. (2016). The King-Devick test of rapid number naming for concussion detection: Meta-analysis and systematic review of the literature. Concussion.

[13] Heitger M.H., Jones R., MacLeod A.D., Snell D.L., Frampton C.M., Anderson T.J. (2009). Impaired eye movements in post-concussion syndrome indicate suboptimal brain function beyond the influence of depression, malingering or intellectual ability. Brain.

[14] King D., Hume P., Gissane C., Clark T. (2015). Use of the King–Devick test for sideline concussion screening in junior rugby league. J. Neurol. Sci..

[15] Galetta K.M., Barrett J., Allen M., Madda F., Delicata D., Tennant A.T., Branas C.C., Maguire M.G., Messner L.V., Devick S. (2011). The King-Devick test as a determinant of head trauma and concussion in boxers and MMA fighters. Neurology.

[16] Tjarks B.J., Dorman J.C., Valentine V.D., Munce T.A., Thompson P.A., Kindt S.L., Bergeron M.F. (2013). Comparison and utility of King-Devick and ImPACT^®^ composite scores in adolescent concussion patients. J. Neurol. Sci..

[17] Leong D.F., Balcer L.J., Galetta S.L., Liu Z., Master C. (2014). The King-Devick test as a concussion screening tool administered by sports parents. J. Sports Med. Phys. Fit..

[18] Chrisman S., Quitiquit C., Rivara F.P. (2013). Qualitative Study of Barriers to Concussive Symptom Reporting in High School Athletics. J. Adolesc. Health.

[19] Meehan W., d’Hemecourt P., Collins C., Comstock R. (2011). Assessment and management of sport-related concussions in United States high schools. Am. J. Sports Med..

[20] McCrory P., Meeuwisse W., Aubry M., Cantu R., Dvořák J., Echemendia R., Engebretsen L., Johnston K., Kutcher J.S., Raftery M. (2013). Consensus statement on concussion in sport: The 4th International Conference on Concussion in Sport held in Zurich, November 2012. Br. J. Sports Med..

[21] King D., Gissane C., Hume P., Flaws M. (2015). The King–Devick test was useful in management of concussion in amateur rugby union and rugby league in New Zealand. J. Neurol. Sci..

[22] Alsalaheen B., Haines J.L., Yorke A., Diebold J. (2015). King-Devick Test reference values and associations with balance measures in high school American football players. Scand. J. Med. Sci. Sports.

[23] Vartiainen M.V., Holm A., Peltonen K., Luoto T., Iverson G., Hokkanen L. (2015). King-Devick test normative reference values for professional male ice hockey players. Scand. J. Med. Sci. Sports.

[24] Oride M.K.H., Marutani J.K., Rouse M.W., Deland P.N. (1986). Reliability study of the Pierce and King-Devick saccade tests. Am. J. Optom. Physiol. Optics..

[25] Rist B., Cohen A., Pearce A. (2017). King-Devick performance following moderate to high exercise intensity bouts. Int. J. Exerc. Sci..

[26] Cohen J. (1988). Statistical Power Analysis for the Behavioural Sciences.

[27] Hopkins W.G., Marshall S.W., Batterham A.M., Hanin J. (2009). Progressive Statistics for Studies in Sports Medicine and Exercise Science. Med. Sci. Sports Exerc..

[28] Silverberg N.D., Luoto T., Ohman J., Iverson G. (2014). Assessment of mild traumatic brain injury with the King-Devick Test® in an emergency department sample. Brain Inj..

[29] Lieberman S., Cohen A.H., Rubin J. (1983). NYSOA K-D test. J. Am. Optom. Assoc..

[30] Konynenbelt B., Harris P., Pérez Robles F. (2016). A comparison of performance on the NYSOA King-Devick Test between Mexican and American school-aged children. Optom Vis. Perform..

[31] Anderson H.D., Biely S.A. (2017). Baseline King–Devick scores for adults are not generalizable; however, age and education influence scores. Brain Inj..

[32] Hinton-Bayre A.D. (2015). Normative Versus Baseline Paradigms for Detecting Neuropsychological Impairment Following Sports-Related Concussion. Brain Impair..

[33] Schmidt J., Register-Mihalik J., Milhalik J., Kerr Z., Guskiewicz K. (2012). Identifying impairments after concussion: Normative data versus individualized baselines. Med. Sci. Sport Exerc..

[34] Valovich McLeod T., Bay R., Lam K., Chhabra A. (2012). Representative baseline values on the Sport Concussion Assessment Tool 2 (SCAT2) in adolescent athletes vary by gender, grade, and concussion history. Am. J. Sports Med..

[35] Echemendia R.J., Bruce J.M., Bailey C.M., Sanders J.F., Arnett P., Vargas G. (2012). The Utility of Post-Concussion Neuropsychological Data in Identifying Cognitive Change Following Sports-Related MTBI in the Absence of Baseline Data. Clin. Neuropsychol..

[36] Harmon K.G., Drezner J.A., Gammons M., Guskiewicz K., Halstead M., Herring S.A., Kutcher J.S., Pana A., Putukian M., Roberts W.O. (2012). American Medical Society for Sports Medicine position statement: Concussion in sport. Br. J. Sports Med..

[37] Rabinowitz A.R., Arnett P.A. (2012). Reading Based IQ Estimates and Actual Premorbid Cognitive Performance: Discrepancies in a College Athlete Sample. J. Int. Neuropsychol. Soc..

[38] Kaplan G.A., Turrell G., Lynch J.W., Everson S.A., Helkala E.-L., Salonen J.T. (2001). Childhood socioeconomic position and cognitive function in adulthood. Int. J. Epidemiol..

[39] Kontos A.P., Elbin R.J., Covassin T., Larson E. (2010). Exploring Differences in Computerized Neurocognitive Concussion Testing Between African American and White Athletes. Arch. Clin. Neuropsychol..

[40] Covassin T., Elbin R., Kontos A., Larson E. (2010). Investigating baseline neurocognitive performance between male and female athletes with a history of multiple concussion. J. Neurol. Neurosurg. Psychiatry.

[41] Covassin T., Elbin R., Bleecker A., Lipchik A., Kontos A.P. (2013). Are There Differences in Neurocognitive Function and Symptoms Between Male and Female Soccer Players After Concussions?. Am. J. Sports Med..

[42] Zimmer A., Piecora K., Schuster D., Webbe F. (2013). Sport and Team Differences on Baseline Measures of Sport-Related Concussion. J. Athl. Train..

[43] Moran R., Covassin T. (2017). Risk factors associated with baseline King-Devick performance. J. Neurol. Sci..

[44] Raynowska J., Hasanaj L., Zhang I. (2015). Agreement of the spiral-bound and computerized tablet versions of the King-Devick test of rapid number naming for sports concussion. Ann. Sports Med. Res..

[45] Galetta K.M., Brandes L.E., Maki K., Dziemianowicz M.S., Laudano E., Allen M., Lawler K., Sennett B., Wiebe D., Devick S. (2011). The King–Devick test and sports-related concussion: Study of a rapid visual screening tool in a collegiate cohort. J. Neurol. Sci..

[46] Silver D., Brown N., Gissane C. (2018). Reported concussion incidence in youth community Rugby Union and parental assessment of post head injury cognitive recovery using the King-Devick test. J. Neurol. Sci..

